# Reversion of Ceftazidime Resistance in *Pseudomonas aeruginosa* under Clinical Setting

**DOI:** 10.3390/microorganisms10122395

**Published:** 2022-12-02

**Authors:** Qi Liu, Liwen Yin, Xinxin Zhang, Guangbo Zhu, Huimin Liu, Fang Bai, Zhihui Cheng, Weihui Wu, Yongxin Jin

**Affiliations:** 1 State Key Laboratory of Medicinal Chemical Biology, Key Laboratory of Molecular Microbiology and Technology of the Ministry of Education, Department of Microbiology, College of Life Sciences, Nankai University, Tianjin 300071, China; 2 Tianjin Union Medical Center, Nankai University Affiliated Hospital, Tianjin 300100, China

**Keywords:** *P. aeruginosa*, ceftazidime resistance, *ampD*, *ampC*, competitive advantage

## Abstract

*Pseudomonas aeruginosa* is an important nosocomial pathogen which frequently becomes resistant to most antibiotics used in chemotherapy, resulting in treatment failure among infected individuals. Although the evolutionary trajectory and molecular mechanisms for becoming β-lactam resistant have been well established for *P. aeruginosa*, the molecular basis of reversion from β-lactam resistant to susceptible is largely unexplored. In this study, we investigated the molecular mechanisms by which a ceftazidime-resistant clinical strain is converted to a ceftazidime-susceptible isolate under the clinical setting. RNA sequencing and genomic DNA reference mapping were conducted to compare the transcriptional profiles and chromosomal mutations between these two isolates. Our results demonstrate that a gain-of-function mutation in *ampD*, via deletion of a 53 bp duplicated nucleotide sequence, is the contributory factor for the conversion. Furthermore, we show for the first time that AmpD is involved in intraspecies competitiveness in *P. aeruginosa*. We also found that AmpD is not responsible for phenotypic changes between R1 and S2, including growth rate, motilities, pyocyanin, rhamnolipid, and biofilm production. This finding provides novel insights into the alteration of β-lactam sensitivity in *P. aeruginosa* under the clinical setting.

## 1. Introduction

*Pseudomonas aeruginosa* is a clinically important opportunistic human pathogen and is responsible for both acute and chronic infections in vulnerable individuals. It causes a variety of infections, including respiratory tract and urinary tract infections, commonly in patients suffering from cystic fibrosis or in individuals with a compromised immune system [1]. Treatment for infections by *P. aeruginosa* is challenging due to its intrinsic resistance as well as acquired resistance mechanisms to a wide range of antibiotics, which often result in therapeutic failure [1].

β-lactams are first-line antibiotics used to treat DTR (difficult-to-treat) Gram-negative bacteria [2]. Due to their high efficacy and low toxicity, β-lactams are most commonly recommended for the treatment of serious infections by *P. aeruginosa* [3]. However, the development of resistance to β-lactams limits their effectiveness. Several important resistance mechanisms have been reported including: (i) overproduction of the chromosomally encoded β-lactamase (AmpC) [4]; (ii) loss/reduced production of the outer membrane porin OprD [5,6]; (iii) expression of multidrug efflux pumps, such as MexAB-OprM, MexCD-OprJ, and MexXY [4,6]; and (iv) acquisition of the extended-spectrum β-lactamase via horizontal gene transfer [7]. Of note, these resistance mechanisms have been found to contribute to the antibiotic resistance of *P. aeruginosa* in the clinical setting. Importantly, via characterization of clinical isolates from a single patient, evolutionary trajectories and molecular mechanisms for antibiotic resistance have been well established in *P. aeruginosa* [5,8,9,10]. However, the molecular basis underlying the reversion from antibiotic resistance to susceptibility in *P. aeruginosa* is largely unexplored.

In this study, we recovered two clinical *P. aeruginosa* isolates from sputum samples of the same patient with sequelae of craniocerebral trauma. The first isolate was obtained soon after the patient was admitted to the hospital, while the second strain was obtained 48 days later. The first isolate, R1, was ceftazidime resistant, whereas the second isolate, S2, was ceftazidime susceptible. Therefore, our aim was to decipher the molecular mechanisms by which the ceftazidime susceptibility evolved in the clinical setting. Our experimental results demonstrated that a gain-of-function mutation in *ampD*, via deletion of a 53 bp duplicated nucleotide sequence, is the contributory factor for this conversion. Furthermore, we have shown for the first time that AmpD is involved in intraspecies competitiveness in *P. aeruginosa*. This finding provides novel insights into the alteration of β-lactam sensitivity in *P. aeruginosa* in the clinical setting.

## 2. Materials and Methods

### 2.1. Basic Characterization of the Pseudomonas aeruginosa Isolates

Bacterial strains and plasmids used in this study are shown in Table S1. All bacteria were grown at 37 °C in L-broth medium (10 g/L tryptone, 5 g/L yeast extract, and 5 g/L NaCl) or on L-agar plates (L-broth supplemented with 1.5% agar). Ceftazidime-resistant (R1) and ceftazidime-sensitive (S2) *P. aeruginosa* strains used in this study were sequentially isolated from sputum samples of the same patient with sequelae of craniocerebral trauma 48 days apart. The 16S rDNA was amplified by PCR (primers listed in Table S2) and analyzed by sequencing to identify the species of these two strains [11]. Random amplified polymorphic DNA (RAPD) typing was conducted using primers 272, 208, and 241 (Table S2) following an early description [12]. Multilocus sequence typing (MLST) was carried out as described before with minor modifications [13] to determine the allelic profiles of these two isolates. Briefly, chromosomal DNA was extracted from overnight cultures of bacterial cells with a genomic DNA purification kit (Tiangen Biotec, Beijing, China). Using the genomic DNA as a template, the internal fragments of the *aroE, acsA*, *mutL*, *guaA*, *ppsA*, *nuoD*, and *trpE* genes were PCR-amplified and sequenced with specific primers (Table S2) [13]. The sequence type (ST) was obtained for each isolate by submitting the gene sequences and analysis on the website http://pubmlst.org/paeruginosa/ (accessed on 5 June 2021). The minimum inhibitory concentration (MIC) of antibiotics was examined using the two-fold serial dilution method according to a previous description [14]. Susceptibility was determined based on the Clinical and Laboratory Standards Institute guidelines (CLSI 2011–2021).

### 2.2. Plasmid Construction and Gene Editing

To replace the *ampD* gene of the R1 strain with the *ampD* gene of the S2 strain, a 1038 bp DNA fragment containing *ampD* and partial immediately adjacent genes (*ampE* and PA4523) was PCR amplified using genomic DNA of S2 as a template (primers in Table S2), digested with *Hin*dIII and *Eco*RI, and then inserted into a pEX18Tc suicide vector, resulting in pEX18–*ampD*_S2_. Gene replacement in R1 was carried out by conjugal transfer of pEX18–*ampD*_S2_ followed by selection for single crossover and then double crossover, as described previously [15]. The resulting strain R1*ampD*_S2_ was confirmed by PCR amplification and sequencing analysis (primers in Table S2). Strain S2*ampD*_R1_ was generated with similar manipulations.

### 2.3. Genomic DNA Reference Mapping

Genomic DNAs of R1 and S2 were extracted from overnight cultures of bacterial cells using DNA purification kit (Tiangen Biotec, Beijing, China). Genomic DNA reference mapping was carried out by GENEWIZ Life Sciences (Suzhou, China) as described in our previous studies [5,16]. The raw sequence data have been deposited in the NCBI with an accessible number PRJNA863030 (www.ncbi.nlm.nih.gov/sra/?term=PRJNA863030).

### 2.4. RNA Isolation, Reverse Transcription, and Real-Time qPCR

Overnight cultures of bacterial cells were diluted 50-fold into fresh L-broth medium and grown to an OD_600_ of 1.0. Total RNA was extracted with RNAprep Pure Cell/Bacteria Kit (Tiangen Biotec, Beijing, China), and the RNA concentrations were measured using a NanoDrop spectrophotometer (Thermo Scientific, Waltham, MA, USA). cDNAs were then synthesized with random primers and PrimeScript Reverse Transcriptase (Takara, Dalian, China). The cDNAs were mixed with qPCR primers (Table S2), SYBR premix Ex Taq II (Takara, Dalian, China), and ddH_2_O for real-time qPCR. qPCR was carried out in a CFX Connect real-time machine (Bio-Rad, Hercules, CA, USA). A 30S ribosomal protein encoding gene, *rpsL*, was utilized as an internal control.

### 2.5. RNA Sequencing and Data Analysis

Total RNA was purified from the R1 or S2 strain as described above. Library construction, sequencing, and analysis services were conducted by GENEWIZ Life Sciences (Suzhou, China) as described in our early studies [5,16]. The raw RNA sequence data have been deposited in the NCBI under an accessible number PRJNA863024.

### 2.6. Pyocyanin, Rhamnolipid, and Motility Assays

Pyocyanin production was examined as previously described with minor modifications [17]. In brief, overnight bacterial culture was diluted 50-fold in fresh medium and cultivated for 24 h. 1 mL supernatant from each bacterial culture was extracted with 0.5 mL of chloroform. A 0.4 mL sample of the lower organic phase was then re-extracted into 0.3 mL of HCl (0.2 N), and the absorbance was measured at 520 nm with a Varioskan Flash (Thermo Fisher Scientific, Vantaa, Finland). Assays for rhamnolipid production by *P. aeruginosa* were conducted using a M8-based agar medium plate as previously described [16].

Three different media were used for swarming (M9 medium with 2 mM MgSO_4_, 0.4% glucose, and 0.1 mM CaCl_2_), swimming (10 g/L tryptone, 5 g/L NaCl, and 0.3% agarose), and twitching (L-broth medium with 1% agar) motility assays. 1 µL of overnight cultures of *P. aeruginosa* was spotted onto the surfaces of swarming and swimming plates or stabbed into twitching plates. The plates were then incubated at 37 °C for 20 h (swarming motility), 30 °C for 20 h (swimming motility), or 37 °C for 18 h (twitching motility). Images of the plates were taken using imager ChemiDocTM XRS + (Bio-Rad, software version 4.0.0.49710).

### 2.7. Quantification of Biofilm

Biofilm formation was examined as described previously with minor modifications [5]. Overnight cultures of bacterial cells were sub-cultured at 37 °C to an OD_600_ of 1.0 in L-broth. The bacteria were then diluted 1:50 into fresh L-broth, and 150 μL of the suspension was aliquoted into each well of a 96-well plate, followed by cultivation at 37 °C for 48 h. After the bacterial culture was discarded, each well was gently washed three times with ddH_2_O, stained with 0.25% crystal violet for 15 min, washed three times with ddH_2_O, and then dried at 65 °C. 200 μL of eluate [methanol (4): acetic acid (1): ddH_2_O (5)] was added to each well and incubated for 15 min at room temperature. The crystal violet solution was examined at a wavelength of 590 nm with the Varioskan Flash (Thermo Fisher Scientific, Vantaa, Finland).

### 2.8. LL-37 Susceptibility Assay

Overnight cultures of *P. aeruginosa* strains were diluted 1:50 and grown at 37 °C to an OD_600_ of 1.0. The bacterial cells were washed twice with PBS and resuspended in PBS. Then, the bacteria were treated with LL-37 for 4 h at a final concentration of 256 μg/mL. Viable bacterial cell numbers were determined by plating on L-agar plates. Bacterial survival rate% = (live bacterial number with LL-37 treatment) × 100/(initial inoculum bacterial number).

### 2.9. Growth Competition Assay

Overnight cultures of *P. aeruginosa* strains R1 and S2 were diluted 50-fold into fresh L-broth medium. The bacterial cells of R1 and S2 were mixed at a ratio of 3:1 and cultured for an additional 7 h with agitation at 200 rpm at 37 °C. For controls, the individual bacterial cells of R1 or S2 were cultured under the same conditions. Then, bacterial cells were serially diluted in physiological saline and plated onto L-agar plates with or without 10 μg/mL piperacillin to enumerate the CFUs of R1 and the total of R1 and S2. The S2/R1 competitive index represents the ratio of recovered bacterial CFU of S2 to that of R1. The competition between R1*ampD*_S2_ and R1 or S2 and S2*ampD*_R1_ was conducted using the same manipulation.

### 2.10. Statistical analysis

GraphPad Prism 7.0 software was used to conduct the statistical analyses. *p* values were calculated using the two-tailed unpaired Student’s *t* test. Difference was considered statistically significant when *p* value was below 0.05.

## 3. Results

### 3.1. Clinical Isolates R1 and S2 Are Clonally Related

**Figure 1:**
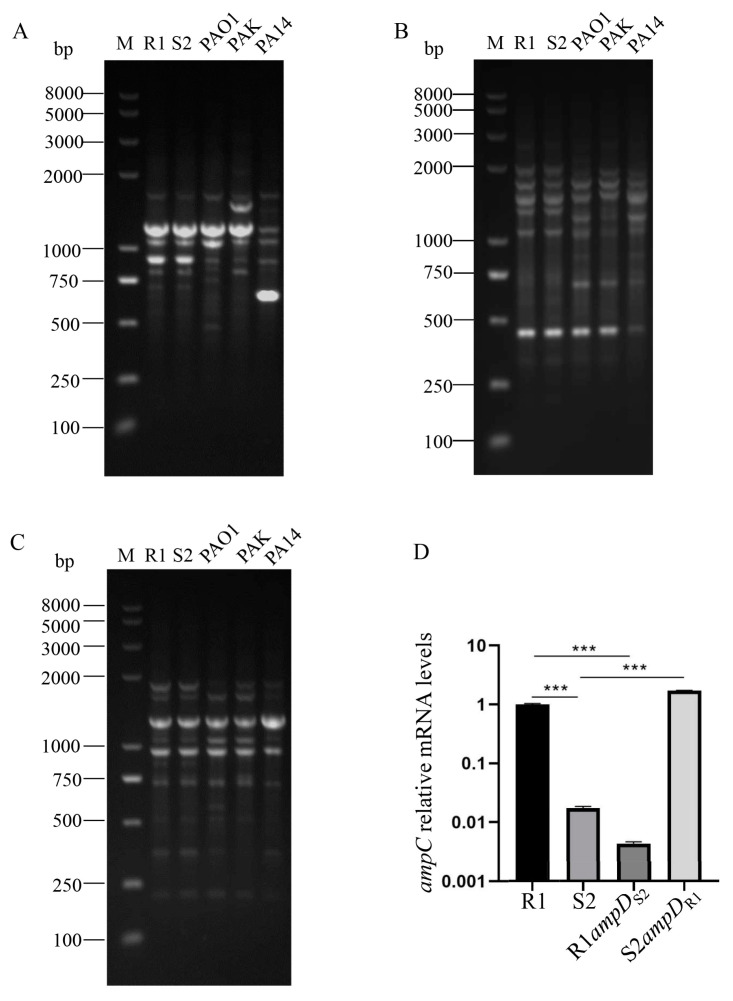
(**A**–**C**) RAPD typing of strains R1 and S2 using primers 272 (**A**), 208 (**B**), and 241 (**C**), M: Marker; (**D**) Relative *ampC* mRNA levels in the indicated bacterial strains. Total RNA was purified from the indicated strains at an OD_600_ of 1.0, and the relative mRNA levels of the *ampC* gene were determined by real-time qPCR using *rpsL* as an internal control, ***, *p* < 0.001, by Student’s *t* test.

Sputum samples from a patient with sequelae of craniocerebral trauma were collected at 48 days apart. Two *P. aeruginosa* strains were isolated, with the first one being resistant to ceftazidime (R1), while the later one being sensitive to ceftazidime (S2). Sequence analysis of the PCR-amplified 16S rDNAs revealed that they both shared 100% identity to that of *P. aeruginosa*. Multilocus sequence typing (MLST), based on sequence analysis of PCR amplicons and genomic DNA reference mapping, demonstrated that R1 and S2 belonged to the same sequence type (ST) of ST-3078 (allelic profiles for *aroE*, *acsA*, *guaA*, *nuoD*, *mutL*, *ppsA,* and *trpE* were 4, 18, 134, 1, 33, 6, and 4, respectively). RAPD typing for the R1 and S2 isolates also demonstrated that they were of the same clonal lineage (Figure 1A–C). Further comparison of R1 and S2 strains via genomic DNA reference mapping revealed a total of 281 SNVs (single nucleotide variations) between the two isolates. In combination, these results suggested that the R1 and S2 isolates are clonally related [1].

### 3.2. Isolate R1 Is Resistant, While S2 Is Susceptible to Ceftazidime

Ceftazidime is a clinically effective β-lactam and is frequently used to treat *P. aeruginosa* infections [18]. Preliminary examination with a VITEK automatic microbe analysis instrument revealed a differential susceptibility against ceftazidime between the R1 and S2 strains. Therefore, we further examined their susceptibility to ceftazidime with the two-fold serial dilution method [14]. As displayed in Table 1, according to the CLSI guidelines, the initial isolate (R1) was resistant to ceftazidime, with an MIC of 32 µg/mL, while the later isolate (S2) displayed susceptibility to ceftazidime, with an MIC of 4 µg/mL, representing an eight-fold decrease. Piperacillin, another β-lactam antibiotic, was also tested. Similar to the ceftazidime, R1 was resistant to piperacillin while S2 was susceptible, with a sixteen-fold difference in the MIC of piperacillin (Table 1).

### 3.3. ampC Is Downregulated in the S2 Strain

To elucidate the molecular mechanism of increased susceptibility to ceftazidime in isolate S2, RNA sequencing was carried out, and the global transcriptional profiles were compared between strains R1 and S2. As shown in Table S3, the expression levels of 68 genes were significantly altered between the two strains. Among them, the β-lactamase-encoding *ampC* gene displayed a 265-fold decreased mRNA level in S2 compared to that in R1. To confirm the observation, we further examined and compared the relative mRNA levels of *ampC* between R1 and S2 by real-time qPCR. Agreeing with the RNA-seq data, the relative mRNA level of *ampC* showed a significant decrease in strain S2 compared to R1 (Figure 1D). To further investigate whether the reduced *ampC* expression contributed to the increased susceptibility to ceftazidime in S2, the *ampC* gene was overexpressed on a plasmid (pUCP24) and introduced into the S2 strain background. Based on the MIC of ceftazidime (Table 1), overexpression of the *ampC* gene reduced the susceptibility of S2 to ceftazidime, with a 16-fold increase in the MIC. These results indicated that decreased expression of the *ampC* gene indeed contributes to the increased susceptibility of S2 to ceftazidime.

### 3.4. A 53 bp Duplicated Nucleotide Deletion in ampD Contributes to the Decreased Expression of ampC and Increased Susceptibility to Ceftazidime in S2

To further explore the mechanism for the increased susceptibility to ceftazidime and the decreased expression of the *ampC* gene in S2, genomic DNA reference mapping was conducted to identify mutations in the genome of S2 relative to that of the R1 strain using the model strain PAO1 as a reference genome (www.pseudomonas.com, accessed on 8 July 2021). Between strains R1 and S2, there were three genes with frameshifts (insertions/deletions) or early stops, fifteen genes had non-synonymous SNVs, and four genes had both frameshifts and non-synonymous SNVs (Table S4). Among them, an *ampD* gene, encoding N-acetyl-anhydromuramyl-L-alanine amidase, which is involved in inducible *ampC* expression and β-lactam antibiotic resistance [19], showed a major difference. In the R1 strain, a 53 bp duplicated nucleotide sequence, corresponding to the 29th–81st nucleotide acids of the *ampD* gene in PAO1, was inserted between the 81st and 82nd base site, resulting in a frameshift after the 27th amino acid of AmpD. PCR amplification and sequencing analysis of the *ampD* gene further validated the genomic DNA reference mapping result. These data suggested that AmpD in R1 is likely inactive.

AmpD inactivation had been found to be the most frequent mechanism resulting in *ampC* hyperexpression and β-lactam resistance in clinical isolates of *P. aeruginosa* [20,21]. The AmpD protein in S2 is identical to that of the PAO1 model strain, while the 53 bp nucleotide frameshift insertion in R1 created a possible inactive AmpD. To assess whether the alteration of *ampD* contributed to the decreased expression of *ampC*, as well as the increased susceptibility to ceftazidime in the S2 strain relative to the R1 strain, the chromosomal *ampD* gene of the R1 and S2 strains was replaced by the *ampD* gene of S2 and R1 using homologous recombination, respectively. As shown in Figure 1D, replacement of *ampD*_S2_ with *ampD*_R1_ increased the mRNA level of *ampC* in the S2 strain, while replacement of *ampD*_R1_ with *ampD*_S2_ in R1 led to a significant decrease in *ampC* transcription (Figure 1D). Consistent with the significantly altered expression of the *ampC* gene, the R1*ampD*_S2_ strain displayed the same MIC against ceftazidime as the S2 strain, while S2*ampD*_R1_ showed the same MIC of ceftazidime as the R1 strain (Table 1). These results demonstrated that AmpD of R1 is nonfunctional and thus unable to repress the expression of *ampC*. However, a gain-of-function mutation in *ampD* is responsible for the decreased expression of *ampC* and increased susceptibility to ceftazidime in the S2 strain.

### 3.5. The S2 Strain Has a Higher Growth Rate and Competitive Advantage over R1

**Figure 2:**
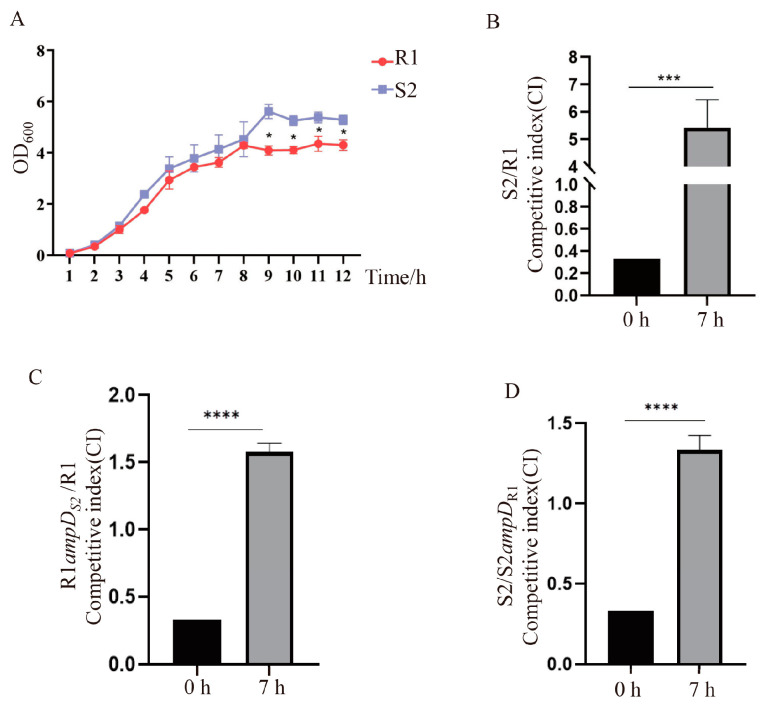
Fitness advantage of the S2 strain compared to the R1 strain. (**A**) Growth curves of R1 and S2 strains in L-broth medium. *, *p* < 0.05, by Student’s *t* test. (**B**–**D**) Competitive advantages of S2 over R1 (**B**), R1*ampD*_S2_ over R1 (**C**), and S2 over S2*ampD*_R1_ (**D**). Two bacterial strains, R1 and S2, R1 and R1*ampD*_S2_, or S2*ampD*_R1_ and S2, were each mixed at a 3:1 ratio, co-incubated for 7 h at 37 °C, and plated on L-agar plates with or without piperacillin. The competitive indexes in (**B**–**D**) represent the ratio of recovered bacterial number (S2, R1*ampD*_S2_, or S2) to the respective number of recovered R1, R1, or S2*ampD*_R1_ strains. Bars represent the means from three tests, and error bars indicate the standard deviation. ***, *p* < 0.001, ****, *p* < 0.0001, by Student’s *t* test.

It has been predicted that in the absence of antibiotic stress, the fitness cost of maintaining resistance would select for loss of the resistance allele, consequently resulting in re-sensitization [22]. Therefore, we examined the growth curves of the R1 and S2 strains. As shown in Figure 2A, the S2 strain displayed an increased growth rate after 9 h compared to the R1 strain. However, R1*ampD*_S2_ and S2*ampD*_R1_ exhibited similar growth rates as their corresponding R1 and S2 parent strains, suggesting that the increased growth rate in S2 was not due to the alteration of *ampD* (Figure S1). This result is consistent with previous studies which reported that inactivation of *ampD* had no effect on the fitness of *P. aeruginosa* due to the presence of two additional *ampD* homologs, *ampDh2* and *ampDh3* [23,24]. However, the absence of all three AmpD amidases severely compromised the growth rates of *P. aeruginosa* [24].

Furthermore, we compared the competitiveness between the R1 and S2 strains. R1 was co-cultured with S2 with a 3:1 ratio of CFU in L-broth for 7 h, and the number of the population of R1 and S2 was determined by enumerating the CFU on L-agar medium with or without 10 μg/mL piperacillin. Piperacillin was used for selection due to the larger difference in MIC between the R1 and S2. We found that the S2 strain had a higher competitive advantage over the R1 strain, with a 16.3-fold higher competitive index (Figure 2B). Since the individual culture of S2 under the same conditions showed a 2.0-fold higher CFU compared to that of R1 (Figure S2A), we reasoned that the competitive advantage was not simply due to the higher growth rate of the S2 strain. Next, we performed competition assays for the R1 and R1*ampD*_S2_ strains, as well as the S2 and S2*ampD*_R1_ strains. As shown in Figure 2C, replacement of the *ampD* in R1 with *ampD*_S2_ rendered a higher competition advantage, with a 4.7-fold increased competitive index. Similarly, replacement of *ampD* in S2 with *ampD*_R1_ resulted in a competitive disadvantage (Figure 2D). No significant difference in individual growth rates between R1*ampD*_S2_ and R1 or S2 and S2*ampD*_R1_ was observed under the same culture conditions (Figure S2B,C).

### 3.6. S2 Displays a Reduced Susceptibility to LL-37 Than R1

**Figure 3:**
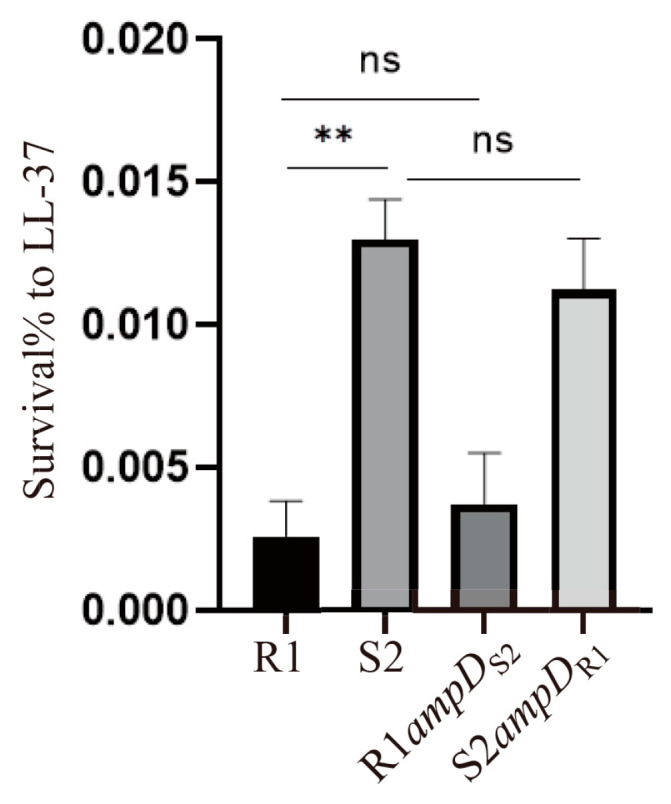
The susceptibility of the indicated bacterial strains to LL-37. *P. aeruginosa* strains were treated with 256 μg/mL LL-37 for 4 h in PBS. The viable bacterial numbers were determined by plating on an L-agar plate. The bacterial survival rate represents the ratio of recovered bacterial CFU following treatment with LL-37 to that of the initial inoculum. The data represent the means from three samples ± standard deviation. ns, not significant; **, *p* < 0.01, by Student’s *t* test.

Antimicrobial peptide LL-37 is the only cathelicidin peptide produced by epithelial cells, macrophages, neutrophils, and lymphocytes of the human body [25,26]. LL-37 exhibits a broad spectrum of antimicrobial activity, functioning as an important first-line defense against pathogenic bacteria [27]. Since S2 might be evolved from R1 in vivo, we reasoned that S2 may have a higher tolerance to LL-37 than R1. To test this hypothesis, we first treated the R1 and S2 strains with 256 μg/mL LL-37 for 4 h in PBS and determined the percentage of viable bacteria by plating and enumerating the CFU on L-agar plates. We found that the S2 strain displayed a significantly higher survival rate in the presence of LL-37 (Figure 3). Replacement of the *ampD*_R1_ with *ampD*_S2_ in the R1 strain resulted in a similar survival percentage in the presence of LL-37 as that of R1. Similarly, replacement of *ampD*_S2_ with *ampD*_R1_ in the S2 strain showed no impact on the survival rate in the presence of LL-37 (Figure 3). These results suggested that the S2 strain has a reduced susceptibility to LL-37, which is not due to the AmpD alteration. Further studies are required to understand the mechanisms resulting in the increased tolerance to LL-37 of the S2 strain.

### 3.7. S2 shows Decreased Motilities and Pyocyanin Production, but Increased Biofilm and Rhamnolipid Production Compared to R1

**Figure 4:**
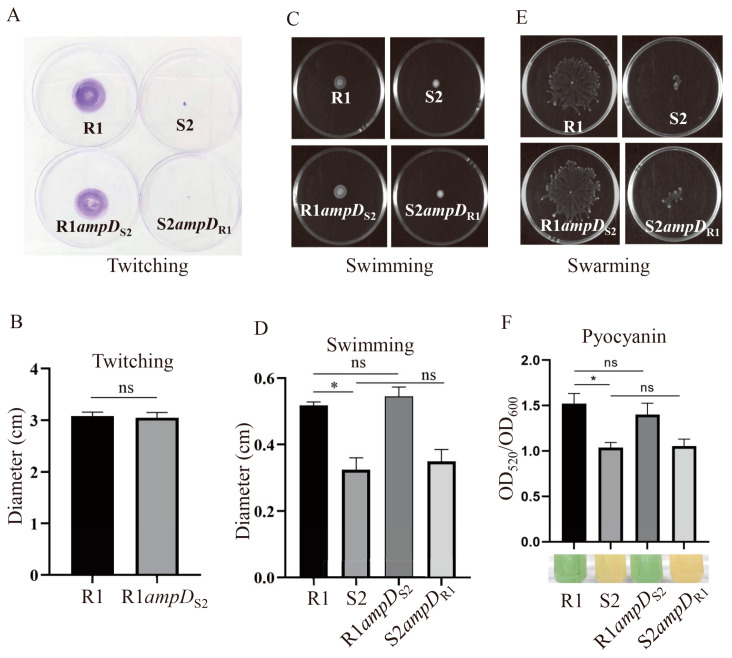
Motility and pyocyanin production were reduced in the S2 strain compared to the R1 strain. Twitching motility on plate (**A**) or diameters of the twitching zone (**B**) for indicated strains. Swimming motility on plate (**C**) or diameters of the swimming zone (**D**) for indicated strains. Swarming motility of the indicated strains on plates (**E**). Pyocyanin production in the indicated strains (**F**). Data presented here represent the means of three samples ± standard deviations. ns, not significant; *, *p* < 0.05, by Student’s *t* test.

**Figure 5:**
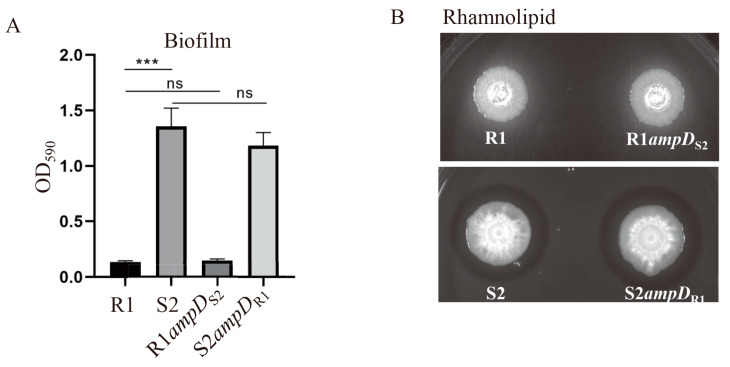
Biofilm formation (**A**) and rhamnolipid production (**B**) by the indicated bacterial strains. (**A**) Cultures of the indicated *P. aeruginosa* strains were diluted 50-fold in L-broth and incubated in each well of a 96-well plate for 48 h at 37 °C. The biofilm in the well was stained with 0.25% crystal violet, dissolved in 200 μL of eluate, and subjected to measurement at OD_590_. ns, not significant; *** *p* < 0.001 by Student’s *t* test. (**B**) Production levels of rhamnolipids were determined on theplate; 1 μL culture of the indicated *P. aeruginosa* strain was plotted onto the plate and incubated at 37 °C for 24 h and then at room temperature for 72 h. The presence of a halo ring around the bacterial colony indicates the production of rhamnolipids.

It has been demonstrated that *P. aeruginosa* commonly shows decreased motilities, enhanced biofilm formation, and altered virulence production to adapt to the environment in the airway of patients with cystic fibrosis [28,29,30,31]. To determine whether the S2 strain acquired phenotypic adaptations relative to the R1 strain, we examined the motilities, production of pyocyanin and rhamnolipid, and biofilm forming abilities of the R1 and S2 strains. As shown in Figure 4, the later isolate S2 displayed impaired swimming, swarming, and twitching motility, whereas the early isolate R1 was proficient in all these motilities. The S2 strain also exhibited significantly decreased production of pyocyanin compared to the R1 strain (Figure 4). Interestingly, the S2 strain was a better biofilm former and rhamnolipid producer than the R1 strain (Figure 5). The above data, in combination with the fact that S2 has a higher growth rate and competitive advantage over R1, as well as the increased tolerance to LL-37, suggests that S2 was likely evolved from strain R1. However, by testing the phenotypes of strains R1*ampD*_S2_ and S2*ampD*_R1_, we found that these phenotypic alterations were not due to the difference in *ampD* between the R1 and S2 strains (Figure 4 and Figure 5).

## 4. Discussion

Antibiotic resistance poses a considerable challenge for the treatment of *P. aeruginosa* infections. Due to their high efficacy and low toxicity, β-lactams are most commonly recommended to treat serious infections by difficult-to-treat *P. aeruginosa*. Understanding the swift and molecular mechanisms of bacterial susceptibility to β-lactams in the clinical setting may provide clues for the development of new or improved therapeutic strategies effective against *P. aeruginosa*. In this study, we identified a gain-of-function mutation in *ampD*, via deletion of a 53 bp duplicated nucleotide sequence, as the contributory factor for the conversion from resistant to susceptible against ceftazidime in a clinical *P. aeruginosa* strain. Of note, our study also indicated that recovery of susceptibility to β-lactam antibiotics in *P. aeruginosa* may occur during the *P. aeruginosa* infections. Hence, antibiotic susceptibility testing was required for repeated isolates at some intervals for optimal antibiotic therapy.

AmpC cephalosporinase is a chromosomally encoded resistance mechanism in *P. aeruginosa*, whose overproduction confers resistance to most β-lactam antibiotics [4]. *ampD* mutations resulting in *ampC* de-repression and decreased susceptibility to β-lactam antibiotics have been reported in *P. aeruginosa* clinical isolates [19,20,21,32,33]. Mutations in the *ampD* gene with amino acid substitutions [32,33], frameshift deletions [21,32], in-frame deletions [32], and IS element insertions [20] have been demonstrated to be the molecular mechanisms of AmpC-hyperproduction-mediated β-lactam resistance in clinical isolates of *P. aeruginosa*. Of note, by investigating a collection of isogenic ceftazidime-susceptible and -resistant pairs of strains, each pair sequentially isolated from a patient treated with β-lactams, Juan et al., found that inactivating mutations of *ampD*, including frameshift mutations, premature stop, and gene deletions, were responsible for AmpC hyperproduction and ceftazidime resistance [21]. In the current study, we sequentially recovered a pair of ceftazidime-resistant and -susceptible *P. aeruginosa* isolates from a single patient and found that a gain-of-function in AmpD, via deletion of a 53 bp duplicated nucleotide, was the molecular mechanism for the decreased *ampC* expression and increased ceftazidime susceptibility. Since the 53 bp inserted nucleotide acid between 81st and 82nd base sites in the R1 strain was a duplication of the 29th–81st nucleotide sequence of the *ampD* gene, we speculate that this fragment in R1 was gained during genome duplication. Similarly, during genome duplication, it might have been lost in the S2 strain. To the best of our knowledge, this is the first time reporting this nucleotide fragment duplication in the *ampD* gene in *P. aeruginosa* clinical strains. Interestingly, this is not the first report of a gain-of-function mutation in *P. aeruginosa* clinical strains. Gain-of-function mutations in the LysR family transcriptional regulator MexT result in constitutive overproduction of the efflux pump MexEF-OprN and increased resistance of *P. aeruginosa* clinical isolates to fluoroquinolones, chloramphenicol, and trimethoprim [34,35]. Gain-of-function mutations in the two-component regulatory system sensor kinase PmrB have been observed to promote polymyxin resistance of *P. aeruginosa* isolates from cystic fibrosis patients [36]. In addition, gain-of-function mutations in the LysR family transcriptional regulator have already been found to constitutively activate the transcription of a complex regulon for aromatic compound degradation in *Acinetobacter baylyi* and the transcription of genes of the cysteine regulon in *Salmonella* enterica serovar *Typhimurium* [37,38].

Our study reveals the function of AmpD in intraspecies competitiveness in *P. aeruginosa.* To the best of our knowledge, this is the first study to link the amidase AmpD with the intraspecies competition of *P. aeruginosa*. However, whether the AmpD alteration is the only contributor to the competitiveness advantage for S2 and the underlying mechanisms of AmpD-mediated competitiveness in *P. aeruginosa* remain elusive and warrant further studies.

Our study demonstrates that S2 displays decreased motilities and pyocyanin production, but increased biofilm and rhamnolipid production compared to the R1, while these phenotypic changes were not caused by the *ampD* mutation. Notably, simultaneous mutation of *ampD* and *ampDh3* had wild-type swimming, twitching, and swarming, while simultaneous inactivation of the three amidases (AmpD, AmpDh2, and AmpDh3) had a substantial effect on twitching, swimming, and swarming in the *P. aeruginosa* PAO1 and PA14 background [24]. By analyzing the RNA-seq results, no difference in the expression levels of genes related to motility, pyocyanin production, biofilm formation, and rhamnolipid production was observed between the R1 and S2 strains (Table S3). Interestingly, genomic DNA reference mapping revealed mutations in the genes *flgK*, *pilB*, *pilY1,* and *pslA* of S2 compared to R1, which were reported to be involved in motility and biofilm formation [39,40,41,42]. Efforts are underway to determine the underlying mechanisms resulting in phenotypic changes in motility and biofilm formation in S2 in comparison to the R1 strain.

Pyocyanin and motility phenotypes (swimming, twitching, and swarming) are considered to be important virulence factors for acute infections, while biofilm formation is important for chronic infections [29]. *P. aeruginosa* undergoes evolutionary adaptations during the chronic infection process, including the loss of motility, reduced production of virulence factors, transition to a biofilm lifestyle, and evolution to a high-level antibiotic resistance [28,29]. Our later isolate S2 exhibited decreased pyocyanin production, impaired motilities (swimming, swarming, and twitching), and increased biofilm production. However, the S2 strain displayed an increased susceptibility to β-lactam antibiotics. Although we did not know the exact medical record of the patient before R1 isolation, the patient did not receive β-lactams in the 48-day period between R1 and S2 isolation. Therefore, we speculate that the R1 bacterial cells encountered a β-lactam-antibiotic-free environment in the host. A similar loss of resistance has been previously observed for polymyxin [36]. Although the molecular mechanisms have been unclear, some highly polymyxin-resistant clinical strains have been observed to gradually lose resistance when they are passaged repeatedly under non-antibiotic selection pressures [36].

In summary, using the sequentially recovered ceftazidime-resistant and ceftazidime-susceptible isolates, we identified that a functional gain of AmpD due to loss of a 53 bp duplicated nucleotide resulted in an increased susceptibility to ceftazidime in the later *P. aeruginosa* isolate S2. We also found that the altered AmpD contributed to the bacterial competition between R1 and S2, but AmpD was not responsible for phenotypic changes between R1 and S2, including growth rate, motilities, pyocyanin, rhamnolipid, and biofilm production.

## Figures and Tables

**Table 1:** MICs (μg/mL) of the indicated *P. aeruginosa* strains.

Strains	Ceftazidime (μg/mL) ^a^	Piperacillin (μg/mL) ^a^
R1	32	128
S2	4	8
R1*ampD*_S2_	4	8
S2*ampD*_R1_	32	128
R1/pUCP24	32	128
S2/pUPC24	4	8
S2/pUCP24-*ampC*	64	256

^a^: Clinical Laboratory Standards Institute (CLSI) susceptibility breakpoints: ceftazidime, ≤8 μg/mL; piperacillin, ≤16 μg/mL; resistance breakpoints: ceftazidime, ≥32 μg/mL; piperacillin, ≥128 μg/mL.

## Data Availability

The raw data of genomic DNA reference mapping have been deposited in the NCBI with an accessible number PRJNA863030. The RNA sequence raw data have been deposited in the NCBI under an accessible number PRJNA863024.

## Supplementary Materials

The following supporting information can be downloaded at: https://www.mdpi.com/article/10.3390/microorganisms10122395/s1, Figure S1. Growth curves of strains R1 and R1*ampD*_S2_ (A) and S2 and S2*ampD*_R1_ (B) in L-broth medium; Figure S2. Growth of individual strains R1, S2 (A), R1, R1*ampD*_S2_ (B), and S2, S2*ampD*_R1_ (C) in L-broth medium under otherwise identical competition conditions. ns, not significant; **, *p* < 0.01, by Student’s *t* test; Table S1. Bacterial strains and plasmids used in this study; Table S2. Primers used in this study; Table S3. Transcriptome analysis: differentially expressed genes by RNA sequencing; Table S4. SNVs identified by genomic DNA reference mapping with elimination of SNVs with heterozygous mutations and the same mutation between R1 and S2. References [43,44,45,46] are cited in the supplementary materials.
