# Supplementary material for: Reversion of Ceftazidime Resistance in Pseudomonas aeruginosa under Clinical Setting
Source: Microorganisms. 2022 Dec 2;10(12):2395. doi: 10.3390/microorganisms10122395 (PMC9782964; doi:10.3390/microorganisms10122395)
Supplement: Supplementary file 1 [file microorganisms-10-02395-s001.zip › Fig. S2.pdf]

Fig. S2

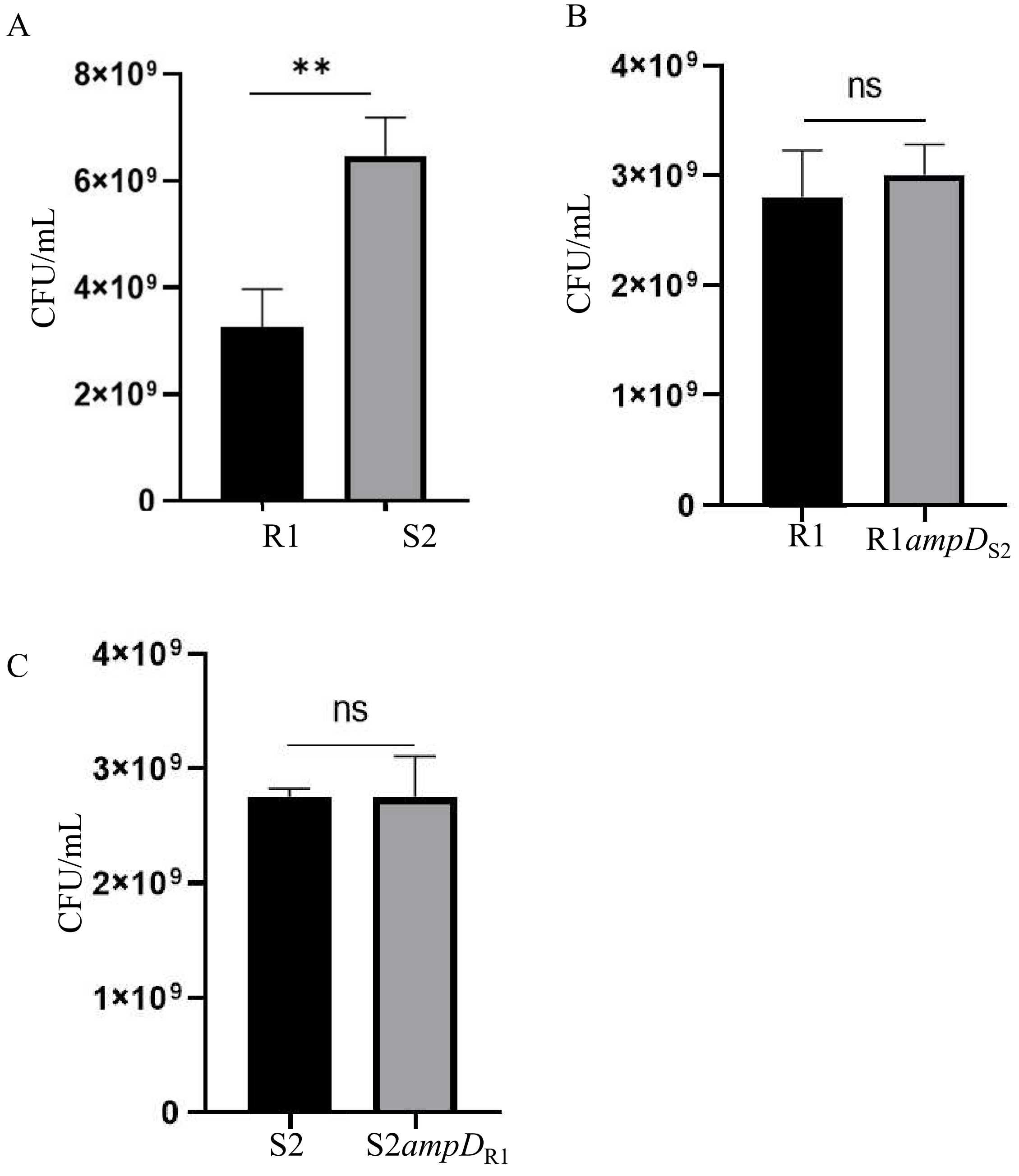

Fig. S2. Individual growth of R1, S2 (A), R1, R1*ampD*$_{S2}$ (B) and S2, S2*ampD*$_{R1}$ (C) strains in L-broth medium under otherwise the competition condition. ns, not significant; **, $p < 0.01$, by Student's t test.
