# Supplementary material for: Reversion of Ceftazidime Resistance in Pseudomonas aeruginosa under Clinical Setting
Source: Microorganisms. 2022 Dec 2;10(12):2395. doi: 10.3390/microorganisms10122395 (PMC9782964; doi:10.3390/microorganisms10122395)
Supplement: Supplementary file 1 [file microorganisms-10-02395-s001.zip › Table S1 strains.docx]

**Table S1.** Bacterial strains and plasmids used in this study.

| Strains or plasmids | | Description | Source or reference |
| --- | --- | --- | --- |
| **strains** | |  |  |
| ***E. coli* strains** | |  |  |
| DH5α | | F^̶^ ϕ 80d*lacZ*∆M15 *endA1 recA1 hsdR17*(r_K_^̶^ m_K_^+^) *supE44 thi-1 relA1* ∆(*lacZYA-argF*) *U169 gyrA96 deoR* | TransGen |
| S17-1 | RP4-2 Tc::Mu Km::Tn*7* Tp^r^ Sm^r^ Pro Res^̶^ Mod^+^ | | Dr. Ramphal |
| ***P. aeruginosa* strains** |  | |  |
| PAO1 | Wild type *P. aeruginosa* strain | | 43 |
| PAK | Wild type *P. aeruginosa* strain | | David Bradley |
| PA14 | Wild type *P. aeruginosa* strain | | 44 |
| R1 | A clinical strain resistant to piperacillin and ceftazidime firstly isolated from a patient in a cooperative hospital | | This study |
| S2 | A clinical strain susceptible to piperacillin and ceftazidime subsequently isolated from the same patient | | This study |
| R1*ampD*_S2_ | *ampD* gene on the R1 genome homologous recombined with *ampD* from S2 | | This study |
| S2*ampD*_R1_ | *ampD* gene on the S2 genome homologous recombined with *ampD* from R1 | | This study |
| **Plasmids** |  | |  |
| pUCP24 | Shuttle vector between *E. coli* and  *P. aeruginosa*; Gm^r^ | | 45 |
| pUCP24-*ampC* | *ampC* gene expressed in pUCP24, Gm^r^ | | 5 |
| pEX18Tc | Gene replacement vector; Tc^r^, *oriT*^+^, *sacB*^+^ | | 46 |
| pEX18-*ampD*_R1_ | *ampD* gene of R1for homologous recombination on pEX18Tc; Tc^r^ | | This study |
| pEX18-*ampD*_S2_ | *ampD* gene of S2 for homologous recombination on pEX18Tc; Tc^r^ | | This study |
