# Supplementary material for: Reversion of Ceftazidime Resistance in Pseudomonas aeruginosa under Clinical Setting
Source: Microorganisms. 2022 Dec 2;10(12):2395. doi: 10.3390/microorganisms10122395 (PMC9782964; doi:10.3390/microorganisms10122395)
Supplement: Supplementary file 1 [file microorganisms-10-02395-s001.zip › Table S2 primers.docx]

**Table S2.** Primers used in this study.

| Primer^a^ | Sequence 5’-3’ | Use | Reference/Source |
| --- | --- | --- | --- |
| *acsA*-F | ACCTGGTGTACGCCTCGCTGAC | Gene amplification for MLST analysis | 13 |
| *acsA*-R | GACATAGATGCCCTGCCCCTTGAT |  |  |
| *aroE*-F | TGGGGCTATGACTGGAAACC |  |  |
| *aroE*-R | TAACCCGGTTTTGTGATTCCTACA |  |  |
| *guaA*-F | CGGCCTCGACGTGTGGATGA |  |  |
| *guaA*-R | GAACGCCTGGCTGGTCTTGTGGTA |  |  |
| *mutL*-F | CCAGATCGCCGCCGGTGAGGTG |  |  |
| *mutL*-R | CAGGGTGCCATAGAGGAAGTC |  |  |
| *nuoD*-F | ACCGCCACCCGTACTG |  |  |
| *nuoD*-R | TCTCGCCCATCTTGACCA |  |  |
| *ppsA*-F | GGTCGCTCGGTCAAGGTAGTGG |  |  |
| *ppsA*-R | GGGTTCTCTTCTTCCGGCTCGTAG |  |  |
| *trpE*-F | GCGGCCCAGGGTCGTGAG |  |  |
| *trpE*-R | CCCGGCGCTTGTTGATGGTT |  |  |
| RAPD primer 272 | AGCGGGCCAA | RAPD analysis  Gene sequencing for MLST analysis | 12 |
| RAPD primer 208 | ACGGCCGACC |  |  |
| RAPD primer 241 | GCCCGAGCGG |  |  |
| *acsA*-SF | GCCACACCTACATCGTCTAT | Gene sequencing for MLST analysis | 13 |
| *acsA*-SR | AGGTTGCCGAGGTTGTCCAC |  |  |
| *aroE*-SF | ATGTCACCGTGCCGTTCAAG |  |  |
| *aroE*-SR | TGAAGGCAGTCGGTTCCTTG |  |  |
| *guaA*-SF | AGGTCGGTTCCTCCAAGGTC |  |  |
| *guaA*-SR | GACGTTGTGGTGCGACTTGA |  |  |
| *mutL*-SF | AGAAGACCGAGTTCGACCAT |  |  |
| *mutL*-SR | GGTGCCATAGAGGAAGTCAT |  |  |
| *nuoD*-SF | ACGGCGAGAACGAGGACTAC |  |  |
| *nuoD*-SR | TGGCGGTCGGTGAAGGTGAA |  |  |
| *ppsA*-SF | GGTGACGACGGCAAGCTGTA |  |  |
| *ppsA*-SR | GTATCGCCTTCGGCACAGGA |  |  |
| *trpE*-SF | TTCAACTTCGGCGACTTCCA |  |  |
| *trpE*-SR | GGTGTCCATGTTGCCGTTCC |  |  |
| *ampD-*F | CCGGAATTCCTGCTGGACGATGCCTTGCTG | *ampD* amplification | This study |
| *ampD-R* | CCCAAGCTTCCAGAAGGTGCAGCGGAAGG |  |  |
| *ampD*-VF | ATGCATTTCGATTCCGTTACCG | *ampD* verification | This study |
| *ampD*-VR | CCAGCAGCGACACCGCGTC |  |  |
| qPCR primer |  | qpcr of *ampC* | This study |
| q*ampC*F | GTGATGAAGGCCAATGACA |  |  |
| q*ampC*R | ATAGCTGAAGTAATGCGGTTC |  |  |

a: F, forward; R, reverse; S, sequencing; U, upstream; D, downstream; V, verification.
