# Supplementary figures and images for: Reversion of Ceftazidime Resistance in Pseudomonas aeruginosa under Clinical Setting
Source: Microorganisms. 2022 Dec 2;10(12):2395. doi: 10.3390/microorganisms10122395 (PMC9782964; doi:10.3390/microorganisms10122395)

Fig. S1

A

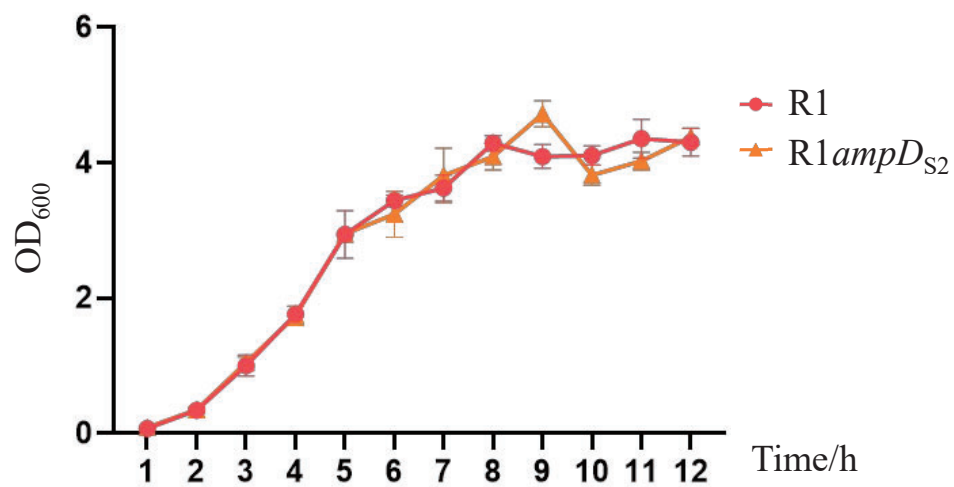

B

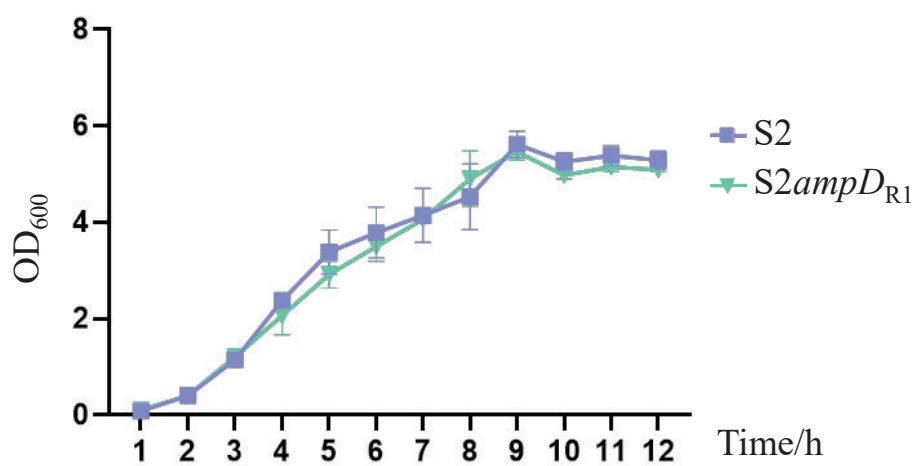

**Fig. S1.** Growth curves of R1, R1ampD<sub>S2</sub> (A) and S2, S2ampD<sub>R1</sub> (B) strains in L-broth medium.

Supplement: Supplementary file 1 [file microorganisms-10-02395-s001.zip › Fig. S1.pdf]
